# Determinants of Impaired Left Atrial Hemodynamics in Paroxysmal Atrial Fibrillation: A 4D Flow MRI Study

**DOI:** 10.3390/jimaging12060222

**Published:** 2026-05-25

**Authors:** Hadi Hassan, Omar Hassan, Shuvam Prasai, Fiza Rajput, Julio Garcia

**Affiliations:** 1 Undergraduate Medical Education, Cumming School of Medicine, University of Calgary, Calgary, AB T2N 1N4, Canada; 2 Faculty of Science, University of Calgary, Calgary, AB T2N 1N4, Canada; 3 Faculty of Medicine and Dentistry, University of Alberta, Edmonton, AB T6G 2E1, Canada; 4 Department of Radiology, Cumming School of Medicine, University of Calgary, Calgary, AB T2N 1N4, Canada; julio.garciaflores@ucalgary.ca; 5 Department of Cardiac Sciences, Cumming School of Medicine, University of Calgary, Calgary, AB T2N 1N4, Canada; 6 Stephenson Cardiac Imaging Centre, Libin Cardiovascular Institute, University of Calgary, Calgary, AB T2N 1N4, Canada; 7 Alberta Children’s Hospital Research Institute, Cumming School of Medicine, University of Calgary, Calgary, AB T2N 1N4, Canada

**Keywords:** atrial fibrillation, left atrium, stasis, cardiac MRI, 4D flow MRI, hemodynamics, stroke risk, hematocrit, BMI, imaging biomarkers

## Abstract

Left atrial (LA) blood flow stasis is a major contributor to thromboembolic risk in atrial fibrillation (AF) and can be measured non-invasively using four-dimensional (4D) flow magnetic resonance imaging (MRI), yet factors driving impaired LA hemodynamics in paroxysmal AF remain unclear. In this retrospective study, 107 patients with paroxysmal AF undergoing pre-ablation cardiac MRI were evaluated. LA blood flow stasis was quantified as the proportion of LA voxels with velocity < 0.1 m/s on 4D flow MRI. Multivariable linear regression assessed associations between LA stasis and demographic, cardiac structural and functional, and clinical variables in a complete-case cohort of 88 patients. Mean LA blood flow stasis was 44 ± 13%. After adjustment, higher left atrial volume index, higher hematocrit, and female sex were independently associated with greater LA stasis, while higher left ventricular ejection fraction, higher resting heart rate, and higher body mass index were associated with lower stasis (all *p* < 0.01). Age and diabetes status were not significantly associated with stasis. The final model explained 40.8% of the variance in LA blood flow stasis. These results underscore the multifactorial nature of LA hemodynamic impairment in paroxysmal AF and support integrating clinical and imaging markers to refine thromboembolic risk assessment.

## 1. Introduction

Atrial fibrillation (AF) is a prevalent cardiac arrhythmia affecting over 52 million individuals worldwide [1]. Four-dimensional (4D) flow magnetic resonance imaging (MRI) has emerged as a non-invasive method to visualize hemodynamic perturbations in cardiac conditions, including AF [2,3]. Prior studies have leveraged 4D flow MRI to establish that individuals with AF display increased blood flow stasis in the left atrium (LA) [4]. Blood flow stasis is a key component of Virchow’s triad, contributing directly to thrombogenesis, and is therefore hypothesized to contribute to the increased stroke risk observed in AF [5]. Blood flow stasis measured using 4D flow MRI correlates with the traditional stroke risk stratification tool used in AF, the CHA2DS2-VASc score [4]. As well, prior 4D flow MRI findings indicate that blood flow stasis levels in AF display considerable between-individual variability [2,6]. This supports further investigation into which individual-level factors are associated with increased LA blood flow stasis to guide selection of candidate patients to receive 4D flow MRI and inform stroke risk stratification in the context of AF.

Prior studies have sought to accomplish this objective. For instance, Spartera et al. (2021) demonstrated that decreased left ventricular ejection fraction (LVEF) and increased LA volume correlate with slower LA blood flow velocities, as measured by 4D flow MRI [7]. However, this study only considered patients with persistent AF. While persistent AF is thought to give rise to a greater risk of stroke [8], there is evidence to suggest that individuals with paroxysmal AF have similarly elevated stroke risk [9]. Indeed, individuals with paroxysmal AF still experience aberrant LA hemodynamics, even in sinus rhythm [10,11], which contributes to thromboembolism. This warrants investigation of clinical and demographic risk factors for impaired LA hemodynamics in this specific population.

To this end, Garcia et al. (2020) examined a clinical cohort of patients with paroxysmal AF and discovered that while age was significantly correlated with LA blood flow stasis, LVEF and LA volume were not [12]. Interestingly, more recent evidence suggests that LVEF and LA volume are correlated with LA blood flow stasis, but only in healthy controls, and not in those with AF [13]. Furthermore, Pradella et al. (2025) unexpectedly demonstrated that LA blood flow stasis correlates inversely with body mass index (BMI) and diabetes status within the general population, but this relationship has not yet been investigated in the specific context of paroxysmal AF [14].

Interestingly, Noda et al. (1996) and Obel et al. (2005) have also shown that increased resting heart rate is linked to decreased left atrial appendage (LAA) flow velocity and potentially higher stasis; however, this relationship has not been examined in the LA [15,16]. Additionally, there is prior computational evidence suggesting a common lab marker, hematocrit (HCT), may increase blood flow stasis within the LA [17], but this theoretical mechanism has not yet been validated in a clinical cohort.

Therefore, the present study aimed to integrate individual characteristics (age, sex, BMI), cardiac structural/functional metrics (LA volume, LVEF, heart rate), and clinical parameters (hematocrit, diabetes status) to identify their unique predictive value for LA blood flow stasis in individuals with paroxysmal AF. Based on the prior literature, we hypothesize that several individual, clinical, and cardiac structural factors will show significant associations with LA blood flow stasis in our paroxysmal AF cohort. We expect that older age, higher LA volume, lower LVEF, higher hematocrit, diabetes, and higher resting heart rate will each be associated with increased stasis, while higher BMI will be associated with reduced stasis. Overall, we anticipate that these relationships observed in previous work will also be present in individuals with paroxysmal AF.

## 2. Materials and Methods

### 2.1. Study Population

In total, 120 patients were retrospectively selected for this study using the Cardiovascular Imaging Registry of Calgary (CIROC). The study population included a pre-ablation group composed of patients with paroxysmal AF who were scheduled to undergo pulmonary vein isolation and had a disease duration of more than two years from initial diagnosis. Patients included in this study had a 4D flow MRI scan between 2017–2020. Eligibility criteria for pre-ablation patients included age ≥ 18 years, sinus rhythm at the time of imaging, which was confirmed at the time of the scan, and no more than mild mitral valve regurgitation. Exclusion criteria included cardiomyopathy, complex congenital heart disease, the presence of implantable cardiac devices, severe renal impairment (estimated glomerular filtration rate ≤ 30 mL/min/1.73 m^2^), or any other recognized contraindications to MRI.

**Figure 1:**
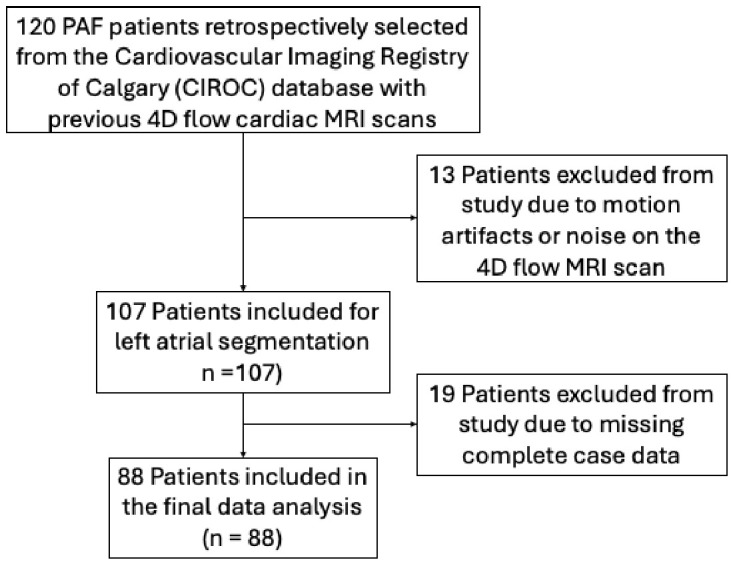
A flowchart diagram of the patients analyzed in this project, following quality control protocols.

Following quality control, which was verified by an experienced CMR reader, 107 patients (n = 107) were included for this study (Figure 1). Thirteen patients were removed due to significant motion artifacts or noise present in the MRI image, hindering our ability to accurately analyze the images.

### 2.2. Cardiac Magnetic Resonance Imaging Protocol

All the cardiac MRI examinations were performed using 3T scanners (Skyra and Prisma, Siemens Healthineers, Erlangen, Germany). A standardized clinical imaging protocol was applied across all participants as previously described [12,18]. Cine images were obtained in standard long-axis views (two-, three-, and four-chamber) as well as contiguous short-axis slices covering the left ventricle.

A gadolinium-based contrast agent (0.2 mmol/kg; Gadovist^®^, Bayer Inc., Mississauga, ON, Canada) was administered intravenously to acquire a contrast-enhanced three-dimensional magnetic resonance angiogram (MRA) of the pulmonary veins for assessment of LA anatomy. Following contrast administration, time-resolved three-dimensional phase-contrast MRI with three-directional velocity encoding (4D flow MRI; Siemens WIP 785A) was performed over approximately 5–10 min to quantify intracardiac blood flow velocities in vivo. This whole-heart 4D flow acquisition protocol has been described previously [12,18].

### 2.3. Conventional Cardiac Image Analysis

Conventional cardiac MRI images were analyzed by medical students under the supervision of an experienced CMR reader. Image analysis was performed using commercially available software (cvi42, Circle Cardiovascular Imaging Inc., Calgary, AB, Canada).

### 2.4. 4D Flow MRI Analysis

Pre-processing of 4D flow MRI datasets included correction for Maxwell terms, eddy current–induced phase offsets, and velocity aliasing when present. For each participant, a three-dimensional phase-contrast MRA was generated and used to perform left atrial segmentation using a proprietary software tool developed in MATLAB (R2024b, MathWorks, Natick, MA, USA). This segmentation included the LAA when present. The segmented LA volume was subsequently applied as a mask to the 4D flow dataset to generate voxel-wise velocity magnitude and stasis maps.

To assess interobserver reproducibility, LA segmentation and stasis quantification were performed independently by two trained analysts under the supervision of an experienced CMR reader. The dataset was initially divided and analyzed in a blinded manner, then fully exchanged and reprocessed by the opposing analyst. Agreement was evaluated based on LA stasis values, with differences < 5% considered acceptable.

**Figure 2:**
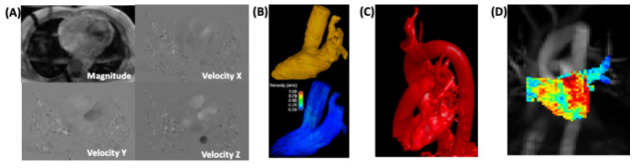
Data processing and analysis workflow: (**A**) Example of 4D-flow MRI data after application of correction steps. (**B**) Depiction of intracardiac flow patterns throughout the full heart volume. (**C**) A time-averaged phase-contrast (PC) magnetic resonance angiography (MRA) dataset is used to define left atrial (LA) anatomy, with a selected intensity threshold to enhance structural visibility; the LA is manually delineated across consecutive slices to obtain a 3D volume. (**D**) Stasis within the LA is subsequently quantified and visualized as maximum intensity projections from the sagittal perspective, with warmer colors (red) indicating greater stasis and cooler colors (blue) indicating reduced stasis.

Velocity magnitude values from all voxels within the segmented LA volume across all cardiac phases were compiled into a velocity histogram. Flow stasis was quantified on a voxel-wise basis as the proportion of cardiac phases during which the velocity magnitude was below 0.1 m/s [2,12]. Mean LA stasis was calculated by averaging voxel-wise stasis values across the entire segmented LA volume. Three-dimensional volumetric stasis maps were generated to facilitate qualitative assessment of the spatial distribution of flow stasis. The overall workflow is also visualized in Figure 2, highlighting the velocity map, the PC MRA image, and ultimately the LA stasis maps used for analysis.

Demographic and clinical variables used for correlation were obtained from the CIROC registry and linked medical records in accordance with institutional research ethics approval.

### 2.5. Statistical Analysis

Continuous variables are presented as mean ± standard deviation. Relationships between demographic and clinical characteristics and blood flow stasis were evaluated using multivariable linear regression analysis. Multivariable linear regression analyses were performed using complete-case data for included predictors, as some variables contained missing values. Predictor variables were specified a priori on the basis of established clinical relevance and evidence from prior studies, as explained in the introduction. These variables were predefined in a single multivariable linear regression including age, BMI, heart rate (HR), LVEF, left atrial volume indexed to body surface area (LAVi), HCT, sex (binary) and diabetes status. Analyses used complete-case data (listwise deletion) such that all included cases had non-missing values for the outcome and all predictors (final N = 88).

All the linear regression assumptions were systematically evaluated. The linearity of associations between independent variables and blood flow stasis was assessed using scatterplots and constant variance of residuals, or homoscedasticity, was assessed using a residual-versus-fitted plot. Independence of observations was ensured through the study design and confirmed with a Durbin-Watson test statistic of 2.44, indicating no evidence of significant autocorrelation. Residual normality was assessed using a quantile–quantile plot in conjunction with the Shapiro–Wilk test, which was not statistically significant. Multicollinearity among predictors was evaluated using variance inflation factors, which were all below 1.5.

No significant deviations from model assumptions were observed. The regression results are reported as unstandardized β coefficients with corresponding 95% confidence intervals. A two-sided *p*-value < 0.05 was considered statistically significant. All statistical analyses were conducted using IBM SPSS Statistics, version 29 (IBM Corp., Armonk, NY, USA).

## 3. Results

### 3.1. Patient Demographics

Patient demographic and clinical characteristics for the pre-ablation study cohort are summarized in Table 1 and are additionally reported here as mean ± standard deviation. The mean age of the study population was 60 ± 10 years. Sex distribution included 70 males and 37 females, corresponding to a female prevalence of 35%. The mean body mass index (BMI) was 29. ± 5.21 kg/m^2^, and the mean resting heart rate was 63 ± 12.9 beats per minute.

Left ventricular ejection fraction (LVEF) was available for 105 patients and demonstrated a mean value of 60 ± 7.04%. Indexed left atrial volume was quantified in 101 patients and averaged 42 ± 13.8 mL/m^2^. Hematocrit measurements, also available for 101 patients, showed a mean value of 0.44 ± 0.04 L/L. Glycated hemoglobin (HbA1c) levels were measured in 93 patients, with a mean HbA1c of 5.68 ± 0.58%.

Based on the Diabetes Canada criteria for HbA1c classification [19], 78 patients were classified as non-diabetic, 9 patients met criteria for prediabetes, and 6 patients were classified as having diabetes mellitus.

### 3.2. Clinical Correlations

**Figure 3:**
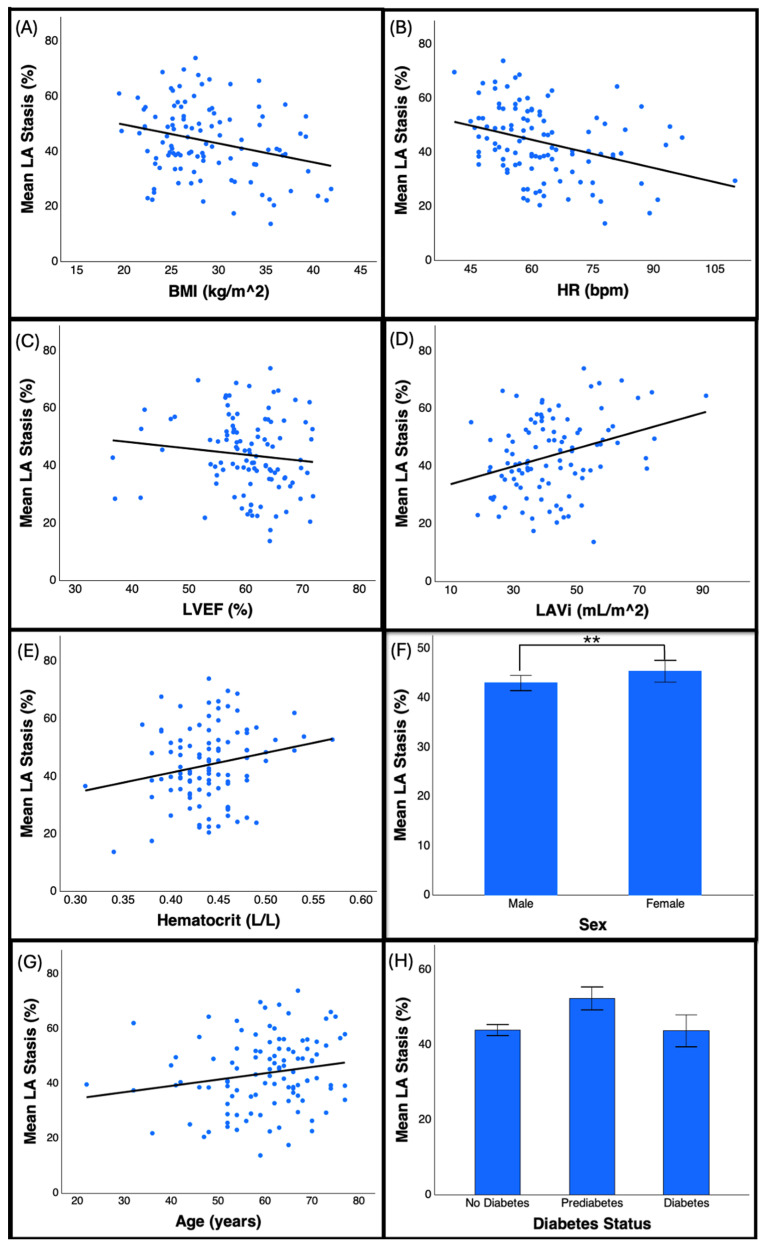
Relationships between clinical measurements and the presence of left atrial stasis. Panels (**A**–**E**) display how body mass index (BMI), resting heart rate (HR), left ventricular ejection fraction (LVEF), left atrial volume index (LAVi), and hematocrit vary in relation to left atrial stasis. Each plot includes a linear regression line, and all observed relationships reached statistical significance after correcting for multiple predictors in a linear regression (*p* < 0.01). Panel (**F**) illustrates the differences in left atrial stasis between male and female patients. Panels (**G**,**H**) illustrate variations in left atrial blood flow stasis by age or diabetes status, neither of which was a significant predictor in the linear regression model. Values are shown as mean ± SE; ** *p* < 0.01.

A total of 107 participants were analyzed in the study. Mean LA stasis for these pre-ablation patients was 43.8 ± 13.12%. Because some variables contained missing values, multivariable linear regression analyses were performed using complete-case data, N = 88, as summarized in Table 2. After adjustment for relevant covariates, multiple demographic and clinical characteristics demonstrated independent associations with mean blood flow stasis (Table 2). These statistically significant correlations are additionally visualized on the scatter plots shown in Figure 3.

**Figure 4:**
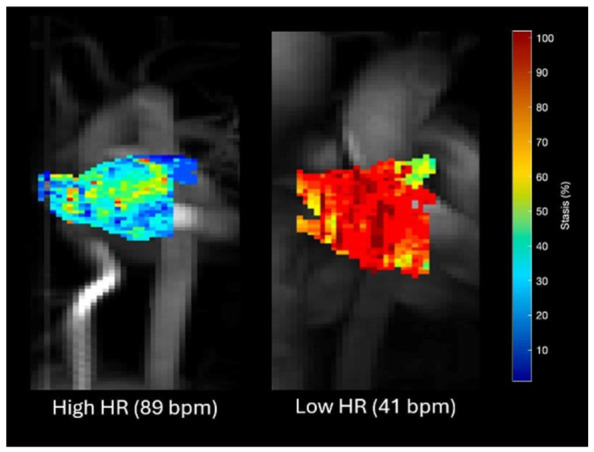
Visualization of heart rate–associated left atrial stasis patterns in a patient with low heart rate and a patient with high heart rate with paroxysmal atrial fibrillation using 4D Flow MRI. Segmented 3D reconstructions of the left atrium, overlaid with a heat map representing voxel-level stasis intensity. The accompanying color scale (0–100) quantifies stasis magnitude, with warmer colors reflecting higher stasis.

In particular, greater body mass index (BMI) (B = − 0.61, 95% CI −1.02 to −0.20, *p* = 0.004), elevated heart rate (B = − 0.37, 95% CI −0.56 to −0.18, *p* < 0.001) (Figure 4), and higher left ventricular ejection fraction (LVEF) (B = − 0.56, 95% CI −0.92 to −0.21, *p* = 0.002) were each associated with lower levels of blood flow stasis.

**Figure 5:**
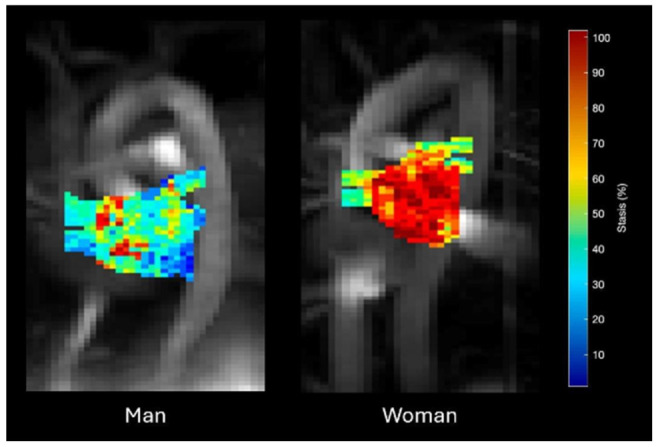
Visualization of left atrial stasis in men and women with paroxysmal atrial fibrillation using 4D Flow MRI. Segmented 3D renderings of the left atrium are shown for male and female participants, with an overlaid heat map representing voxel-wise stasis values. Color intensity corresponds to the stasis scale displayed to the right (ranging from 0–100). Warmer colors indicate regions of higher stasis.

Conversely, increasing LAVi (B = 0.24, 95% CI 0.08 to 0.40, *p* = 0.004) and higher hematocrit values (B = 0.92, 95% CI 0.32 to 1.52, *p* = 0.003) were positively associated with blood flow stasis. Female sex also remained a significant independent predictor, indicating that women displayed higher blood flow stasis (B = 8.62, 95% CI 3.55 to 13.69, *p* = 0.001) (Figure 5).

Interestingly, neither age (B = 0.14, 95% CI −0.08 to 0.36, *p* = 0.20) nor the presence of diabetes mellitus (B = 1.35, 95% CI −2.66 to 5.36, *p* = 0.51) demonstrated a statistically significant association with blood flow stasis after multivariable adjustment. Overall, the final regression model (N = 88) accounted for 40.8% of the variability in blood flow stasis (adjusted R^2^ = 0.408).

## 4. Discussion

The present study aimed to examine the unique associations of demographic and clinical characteristics with LA blood flow stasis in paroxysmal AF using 4D flow MRI. To this end, our investigation revealed that greater LAVi, lower LVEF, female sex, and higher HCT levels were independently associated with greater LA blood flow stasis. Inversely, increasing BMI and HR were independently associated with lower LA blood flow stasis.

LAVi has received considerable attention in the context of AF, as left atrial enlargement is known to be both a consequence of [20] and a risk factor for developing AF [21]. The present study corroborates existing evidence suggesting LA enlargement correlates with greater LA stasis [6,11,13]. As explained by Bäck et al. (2023), the association between LA enlargement and increased LA blood stasis suggests that atrial cardiomyopathy may be a primary contributor to stroke risk in AF [11]. This implies that structural atrial remodelling, rather than the arrhythmia itself, may play a dominant role in thromboembolic risk. Our findings reinforce this claim, as larger LAVi was independently associated with greater LA stasis measured during sinus rhythm.

In addition to LA enlargement, AF-induced cardiomyopathy gives rise to LV systolic dysfunction [22], and reduced LVEF is known to correlate with greater cardioembolic stroke risk [23]. Mechanistically, several studies have demonstrated, using 4D flow MRI, that lower LVEF is associated with aberrant LA hemodynamics in general [13,14] and specifically in AF [6,24]. Our study builds upon these findings by demonstrating that independent of the presently discussed factors, lower LVEF remains a significant predictor of greater LA stasis.

Women with AF are known to experience greater stroke risk and all-cause mortality relative to men [25]. In principle, the fact that women demonstrate larger LAVi than men [26] could explain the increased incidence of thromboembolic complications (e.g., stroke) resulting from AF, as increased LAVi is known to correlate with increased LA blood stasis (as described above) [6,11,13]. However, after controlling for LAVi and other individual parameters, female sex remained independently associated with greater LA stasis in paroxysmal AF. This finding, in line with prior work [24], highlights the need to characterize the sex-specific pathophysiology that gives rise to worse clinical outcomes for women with AF [27].

Emerging evidence suggests that HCT levels are elevated in patients with AF [28,29]. Interestingly, increased HCT has been associated not only with a higher risk of stroke [30] but also with greater stroke severity [31]. These relationships emphasize the importance of hemorheological factors in shaping thromboembolic vulnerability. Prior computational studies have further proposed a mechanistic basis for this association by demonstrating that elevated HCT can increase blood viscosity, reduce flow velocities, and ultimately promote greater blood flow stasis within the LA [17]. The present study provides important clinical support for this concept by validating, in a real-world paroxysmal AF cohort, that higher HCT is independently linked to increased LA stasis even after accounting for structural, functional, and demographic variables. This reinforces the directionality suggested by modeling work, indicating that incremental rises in HCT may possibly contribute to impaired LA washout, although the present correlational analysis does not allow strict determination of causation. By demonstrating that HCT adds unique predictive value beyond conventional imaging parameters, our findings suggest that incorporating HCT into the clinical interpretation of LA flow characteristics may improve individualized assessments of thromboembolic susceptibility in AF and may help guide targeted use of advanced hemodynamic imaging.

Conversely, higher HR independently predicted lower LA stasis. At first glance, this finding appears to differ from earlier TEE-based studies, which reported that increasing HR was associated with reduced LAA emptying velocity and consequently greater LAA stasis [15,16]. However, these investigations specifically examined LAA flow in patients with chronic or non-valvular AF, whereas the present study evaluated global LA hemodynamics in paroxysmal AF during sinus rhythm. This distinction is important. The LAA exhibits markedly different hemodynamic behavior from the LA body [6], and rate-related impairments within the appendage cannot be assumed to mirror patterns across the broader atrial chamber. Additionally, TEE measures instantaneous Doppler-derived velocities at a single anatomical site while 4D flow MRI captures time-averaged, three-dimensional flow patterns throughout the entire LA providing a fundamentally different characterization of atrial washout. These anatomical, physiological, and methodological differences likely account for the opposite directionality observed in our cohort, where higher HR corresponded to lower LA stasis. Future studies employing 4D flow MRI to concurrently assess the LA and LAA in individuals with paroxysmal AF will be essential to clarify whether the association of HR with stasis differs regionally and to determine whether resting HR may still serve as a meaningful clinical marker of impaired intracardiac washout and thromboembolic risk.

Importantly, there is a persistent assumption in healthcare that higher BMI inevitably corresponds to poorer cardiovascular outcomes; however, extensive evidence in AF consistently challenges this view. Large AF cohorts have demonstrated an “obesity paradox,” in which individuals with higher BMI experience lower risks of stroke and mortality. The ARISTOTLE trial reported that overweight and obese patients had significantly reduced all-cause mortality and cardiovascular events relative to normal-weight individuals [32], and the findings from the Gulf SAFE registry similarly showed that obesity was associated with markedly lower risks of stroke or systemic embolism, bleeding, and all-cause mortality [33]. Meta-analytic evidence further reinforces this pattern, demonstrating a graded reduction in thromboembolic risk with increasing BMI across randomized NOAC trials [34]. Despite consistent epidemiologic observations, the physiological basis for this paradox remains unclear. Proposed explanations include differences in body composition not captured by BMI alone, greater metabolic reserve against catabolic stress, variations in cardiorespiratory fitness [35,36], and the possibility that individuals with higher BMI may receive earlier diagnosis or more intensive clinical management [37]. Reverse causation may also contribute, as lower BMI in AF populations can reflect frailty or underlying chronic illness [38,39].

However, these hypotheses largely address confounding and selection bias rather than direct hemodynamic mechanisms that might explain reduced thromboembolic risk. Within the 4D flow MRI literature, Pradella et al. (2025) provided the first indication of a potential mechanistic link by showing that higher BMI independently predicted lower stasis in the LAA, even after accounting for LA volume and other cardiovascular risk factors [14]. Our findings build upon this framework by demonstrating that in patients with paroxysmal AF imaged in sinus rhythm, higher BMI is also independently associated with lower LA stasis. While our retrospective analysis does not allow determination of causation, this finding extends the directionality observed by Pradella et al. 2025 and offers a plausible hemodynamic substrate through which elevated BMI may confer the unexpectedly favorable clinical outcomes documented across diverse AF populations [14].

Notably, after controlling for the above-detailed factors, age and diabetes status were not independently correlated with LA stasis. Age is a well-established risk factor for the development of AF [40] and subsequent stroke risk therein [41]. Prior studies have demonstrated that older age correlates with greater LA stasis [11,12]. However, this effect of age on LA stasis may be mediated by age-related cardiac changes, such as increased LA volume [42], which is known to increase LA stasis as described above [6,11,13]. In support of this theory, the present study demonstrates that when controlling for LA volume, age is no longer a significant independent predictor of LA stasis. Notably, this contrasts with similar work by Pradella et al. (2025), who indeed noted age as an independent predictor of LA stasis (even after controlling for LA volume) [14]. This discrepancy may be explained by the fact that the cohort studied in Pradella et al. (2025) was substantially older (mean age = 73 years) than that of the present study (mean age = 60 years), which may have enabled age effects to be more readily apparent in their sample [14].

Furthermore, while Pradella et al. (2025) unexpectedly demonstrated that diabetes status independently predicts lower LA stasis, the findings from the present study refute this association [14]. However, it is important to note that only a small subset of participants included in the present analysis had prediabetes or diabetes, which limits the statistical power of these conclusions. Future work is needed to clarify the influence of diabetes on LA flow characteristics, given that the presence of diabetes is known to increase stroke risk amongst those with AF [43].

In light of these considerations, the role of 4D flow MRI in this context is best viewed as complementary rather than routine. Specifically, the present findings support the concept that commonly available clinical and imaging variables may help identify individuals with potentially impaired atrial hemodynamics, thereby informing a more targeted approach to advanced imaging. In cases where conventional risk stratification provides an incomplete or uncertain assessment, 4D flow MRI may offer additional physiological insight into patient-specific thromboembolic risk.

## 5. Limitations

This study has a few important limitations that merit consideration. First, the cohort consisted exclusively of individuals with PAF who underwent imaging in sinus rhythm. While this design reduces rhythm-related variability in hemodynamic assessment, it also limits the ability to generalize the findings to patients with persistent or long-standing, persistent AF, who often exhibit a greater arrhythmia burden and more advanced structural remodeling of the LA. Future investigations including and stratifying across AF subtypes would help clarify whether the determinants of stasis identified here are preserved across the broader AF population.

Second, LAA flow was not evaluated separately. Because the LAA is the predominant site of thrombus formation in AF [44] and demonstrates hemodynamic behaviour distinct from the body of the atrium [6], the absence of appendage-specific imaging limits the ability to draw conclusions about this clinically critical structure. Indeed, prior studies have successfully utilized 4D flow MRI to visualize hemodynamics in the LAA [45], and thus future such works should aim to incorporate dedicated LAA assessment to provide a more comprehensive understanding of the mechanisms underpinning thromboembolic risk in AF.

Finally, this was a single-center study, conducted using standardized imaging equipment and acquisition protocols. While this consistency strengthens internal validity, it may limit generalizability to settings with differing patient populations or imaging approaches. Multi-center validation will be important to confirm the broader applicability of these findings.

## 6. Conclusions

In this study of individuals with paroxysmal AF imaged in sinus rhythm, we identified multiple independent clinical, demographic, and structural correlates of LA blood flow stasis quantified using 4D flow MRI. Higher LAVi, HCT, and female sex were associated with increased stasis, whereas higher LVEF, HR, and BMI were associated with reduced stasis. In contrast, age and diabetes status were not independently related to stasis after multivariable adjustment. Together, these factors explained a substantial proportion of inter-individual variability in LA stasis, highlighting the multifactorial determinants of atrial hemodynamics in paroxysmal AF.

These findings extend prior work by demonstrating that aberrant LA flow characteristics persist in paroxysmal AF and are meaningfully associated with readily available clinical and imaging markers. Importantly, the observed association between HCT and stasis provides clinical support for previously proposed hemorheological mechanisms, while sex-specific differences underscore potential biological heterogeneity in atrial flow dynamics. Collectively, this work provides preliminary support for the use of integrated clinical and imaging parameters to better contextualize LA hemodynamic impairment and may inform targeted application of 4D flow MRI and future refinement of stroke risk stratification in PAF. With future validation, these markers may help triage paroxysmal AF patients for 4D flow MRI when conventional risk stratification is equivocal, thereby focusing advanced imaging on those most likely to benefit.

## Figures and Tables

**Table 1:** Demographic and clinical characteristics of PAF patients analyzed in this study.

Characteristic	N	Value
Demographics		
Age, years	107	60 ± 10
Sex, female	107	37 (35%)
Body Mass Index, kg/m^2^	107	29 ± 5
Clinical Variables		
Heart Rate, bpm	107	63 ± 12.9
Hematocrit, L/L	101	0.44 ± 0.04
Diabetes status, % non-diabetic	93	84%
Echocardiographic Variables		
Left Ventricular Ejection Fraction, %	105	60 ± 7.04
Left Atrial Volume Index, mL/m^2^	101	42 ± 13.8
Outcome		
Left Atrial Blood Flow Stasis, %	107	43.8 ± 13.1

**Table 2:** Multivariable linear regression analysis of demographic, clinical, and echocardiographic factors associated with left atrial blood flow stasis. Complete-case data (N = 88). The adjusted R^2^ for the model was 0.408. CI: confidence interval.

Predictor	Unstandardized B	95% CI	Standard Error	Standardized B	*p* Value
Age, years	0.141	−0.077 to 0.359	0.110	0.112	0.202
Sex, female	8.618	3.545 to 13.690	2.548	0.316	0.001
Body Mass Index, kg/m^2^	−0.605	−1.015 to −0.196	0.206	−0.254	0.004
Heart Rate, bpm	−0.370	−0.559 to −0.181	0.095	−0.371	<0.001
Hematocrit, L/L	0.920	0.320 to 1.521	0.302	0.283	0.003
Diabetes status	1.349	−2.567 to 5.355	2.013	0.059	0.505
Left Ventricular Ejection Fraction, %	−0.560	−0.915 to −0.205	0.178	−0.307	0.002
Left Atrial Volume Index, mL/m^2^	0.237	0.077 to 0.397	0.080	0.258	0.004

## Data Availability

The anonymized data presented in this study are available upon reasonable request and a data-sharing agreement. The data is not publicly available due to privacy and ethical restrictions.
